# Echogenic foci with comet-tail artifact in resected thyroid nodules: Not an absolute predictor of benign disease

**DOI:** 10.1371/journal.pone.0191505

**Published:** 2018-01-19

**Authors:** Hongxun Wu, Bingjie Zhang, Jie Li, Qianyun Liu, Tingting Zhao

**Affiliations:** Department of Ultrasound, Jiangyuan Hospital Affiliated to Jiangsu Institute of Nuclear Medicine (Key Laboratory of Nuclear Medicine, Ministry of Health/Jiangsu Key Laboratory of Molecular Nuclear Medicine), Wuxi, Jiangsu, China; Wayne State University, UNITED STATES

## Abstract

The purpose of this study was to evaluate the frequency of echogenic foci with comet-tail artifact in histologically proven thyroid nodules, and to determine the types of echogenic foci with comet-tail artifact that are associated with malignancy. We retrospectively analyzed the sonographic findings of echogenic foci with comet-tail artifact, present in thyroid nodules in 63 patients who underwent surgery for thyroid nodules at our institution between January 2016 and September 2016. The sonographic findings (appearance and background of echogenic foci, shape of comet-tail artifact) in benign and malignant nodules were compared. Seventy-one (7.4%) nodules with ultrasound finding of echogenic foci with comet-tail artifact were encountered in 962 thyroid nodules of 556 patients; 25 of these were benign, and 46 were malignant. Among the echogenic foci with comet-tail artifact categories, those (11/11, 100%) freely distributed in cystic components were all in benign nodules, whereas those (48/67, 71.6%) any part of echogenic foci or comet-tail artifact associated with solid components, were more common in malignant nodules (*P* < 0.001). There was no statistically significant difference in the appearance of echogenic foci and the shape of comet-tail between the benign and malignant nodules (*P* = 0.139, *P* = 0.626, respectively). Echogenic foci with comet-tail artifact freely distributed in cystic component may predict a benign nodule; those associated with solid components cannot be considered a benign finding.

## Introduction

Thyroid carcinoma is a relatively common endocrine malignancy with a favorable prognosis. However, the management remains somewhat controversial. According to American Thyroid Association (ATA) guidelines for thyroid nodules and differentiated thyroid cancer, sonographic evaluation is strongly recommended in patients with suspicious thyroid nodules, nodular goiter, or radiographic abnormality suggesting a thyroid nodule [1]. Known sonographic features suggestive of thyroid malignancy include hypoechogenicity, microcalcification, a taller-than-wider shape and irregular (speculated or microlobulated) margin. Up to 42% of all papillary thyroid carcinomas may show evidence of microcalcification [2], the reported diagnostic specificity of microcalcification for malignant has ranged between 86% and 95% [3–4]. However, it is always inappropriate to identify punctate echogenic foci as microcalcifications by residents, inexperienced/experienced radiologists or non-endocrine specialists in practice. American College of Radiology most recent committee on Thyroid Imaging Reporting and Data System defined echogenic foci as focal regions of markedly increased echogenicity within a nodule relative to the surrounding tissue [5]. Punctate echogenic foci show a spectrum of sonographic appearances which range from bright spots with comet-tail artifact, a benign finding related to cystic colloid nodules, to microcalcification probably corresponding to psammoma bodies at histological examination [5]. Although microcalcification is a highly specific sign of malignancy and is frequently detected in papillary thyroid carcinoma, little consensus exists on the interpretation of echogenic foci with a comet-tail artifact. Echogenic focus with posterior comet-tail artifact, a potential finding indicative of inspissated colloid calcification [6], has been described as a specific benign finding in predominantly cystic nodules [7–9]. However, Malhi *et al*. found that small comet-tail is associated with a relatively high prevalence of malignancy of 15.4% [10]. The significance of echogenic foci with comet-tail artifacts on ultrasonography of thyroid nodules is not well-established. The purpose of this study was to evaluate the frequency of echogenic foci with comet-tail artifact in histologically proven thyroid nodules, and to determine the types of echogenic foci with comet-tail artifact that are associated with malignancy. To our knowledge, this has not been addressed in the published data.

## Materials and methods

Our Institutional Review Board approved this retrospective study and the requirement for consent was waived off. Informed consent was signed and obtained from all patients prior to UG-FNA or surgery.

### Patients

Between January and September 2016, a total of 560 patients underwent thyroid surgery at our institution. These comprised of 427 women (76.25%) and 133 men (23.75%). Mean age of patients was 47.66 years (range, 12–89). The indications for surgery were any one of the following: (1) abnormal results of UG-FNA, including malignancy, suspicion of malignancy, and follicular lesion of undetermined significance; (2) suspicious malignant US findings, including hypoechogenicity, microcalcification, a taller-than-wide shape, and associated cervical lymphadenopathy with round shape, intranodal cystic components, or microcalcification; and (3) pressure symptoms [11]. Four patients for whom complete sonographic data was not available were excluded. Identification of 962 thyroid nodules in 556 patients was achieved via ultrasound imaging, surgical record and histopathological report.

Seventy-one thyroid nodules containing echogenic foci with comet-tail artifact were identified in 63 patients (47 female and 16 male; mean age, 44 years; range, 20–70). Under certain circumstance, echogenic foci in one thyroid nodule with different manifestations were documented separately. Finally, seventy-eight echogenic foci with individual morphological features were documented.

### Ultrasound

All patients underwent routine preoperative ultrasound performed by one of the eight radiologists with an average of 14.88 years (4,5,8,19,19,21,21 and 22 years, respectively) of experience in thyroid imaging. Ultrasound was performed with a 5–12 MHz linear-array transducer (Philips iU22, Philips Healthcare, Bothell, WA, USA). All radiologists selected the presets for small parts and superficial to start evaluation, modified sets of imaging parameters included: Gain: 70; Grayscale mapping: 5; Depth: 4 cm; Frame Rate: 42Hz; Compress: 55; Focal zones: Single; Resolution Penetration: Low; Dynamic Resolution-Speed: Res; 2D Optimization: Res. The following sonographic features were assessed to identify suspicious nodules (hypoechogenicity, taller-than-wide shape, microcalcification, irregular or spiculated/microlobulated margins) or dominant nodules (the largest nodule, if no suspicious nodules were present): size; composition (solid, mixed, cystic, spongiform); echogenicity (marked hypoechoic, hypoechoic, isoechoic, hyperechoic); shape (oval-to-round, irregular, taller-than-wide); margins (smooth, irregular, spiculated, microlobulated); echogenic foci (punctate, macrocalcification, peripheral calcification or comet-tail artifacts) [5] and vascularity (perinodular or intranodular).

At our institution, sonographic assessment of punctate echogenic foci is a standard preoperative procedure since September 2015. Therefore, review of subtypes of punctate echogenic foci can be performed via static images and ultrasound reports. Presence or absence of comet-tail artifact as well as the background of punctate echogenic foci will be described in ultrasound reports. Static images of punctate echogenic foci were captured separately according to their background (Categories are described in the following section).

**Fig 1:**
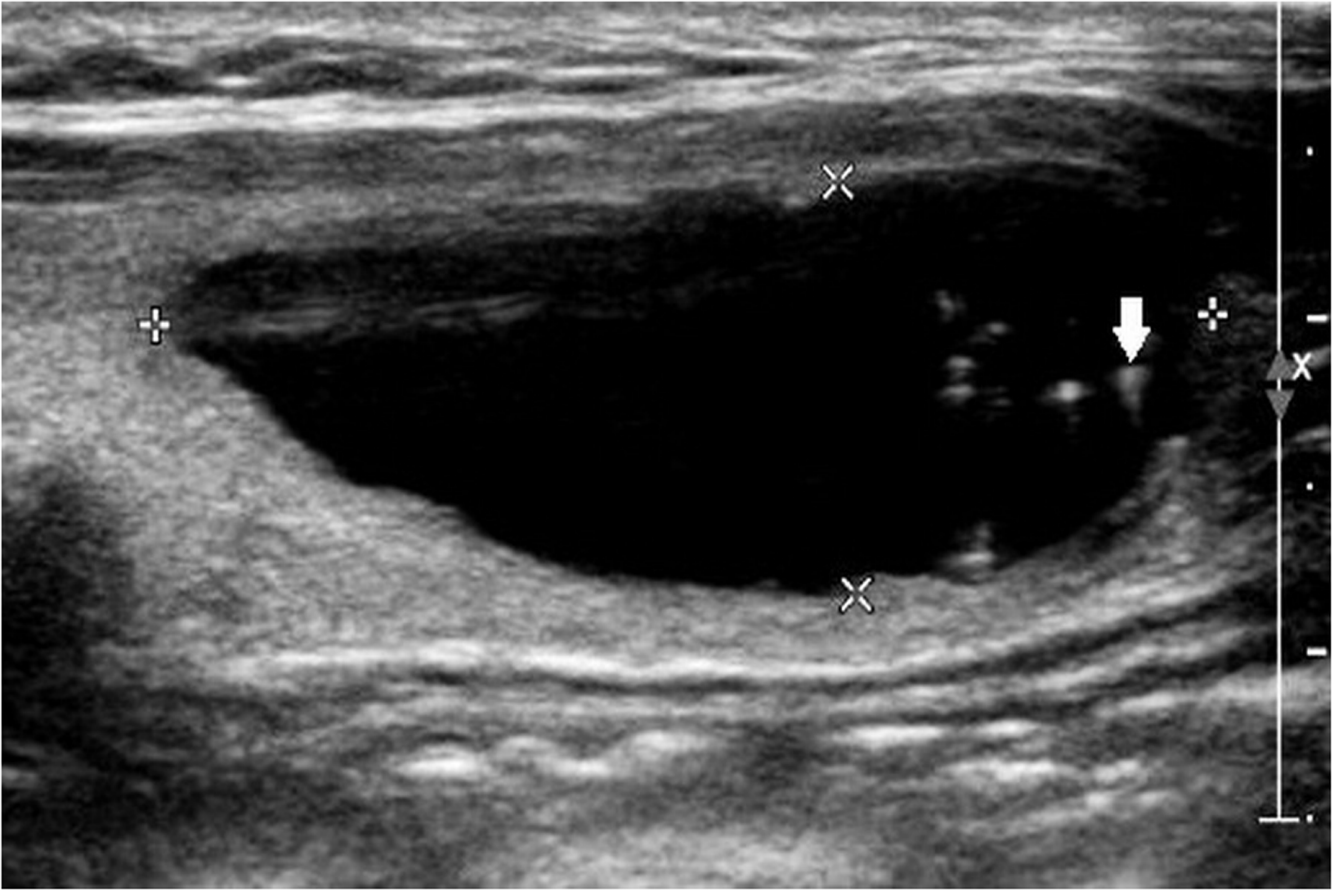
A 46-year-old woman with degenerated multinodular goiter. Longitudinal sonogram shows a 35 mm, smooth, cystic anechoic nodule in the left thyroid lobe. Echogenic foci with comet-tail artifact freely distributed (solid arrow) (Type 1). The patient underwent surgery due to contralateral thyroid malignancy.

**Fig 2:**
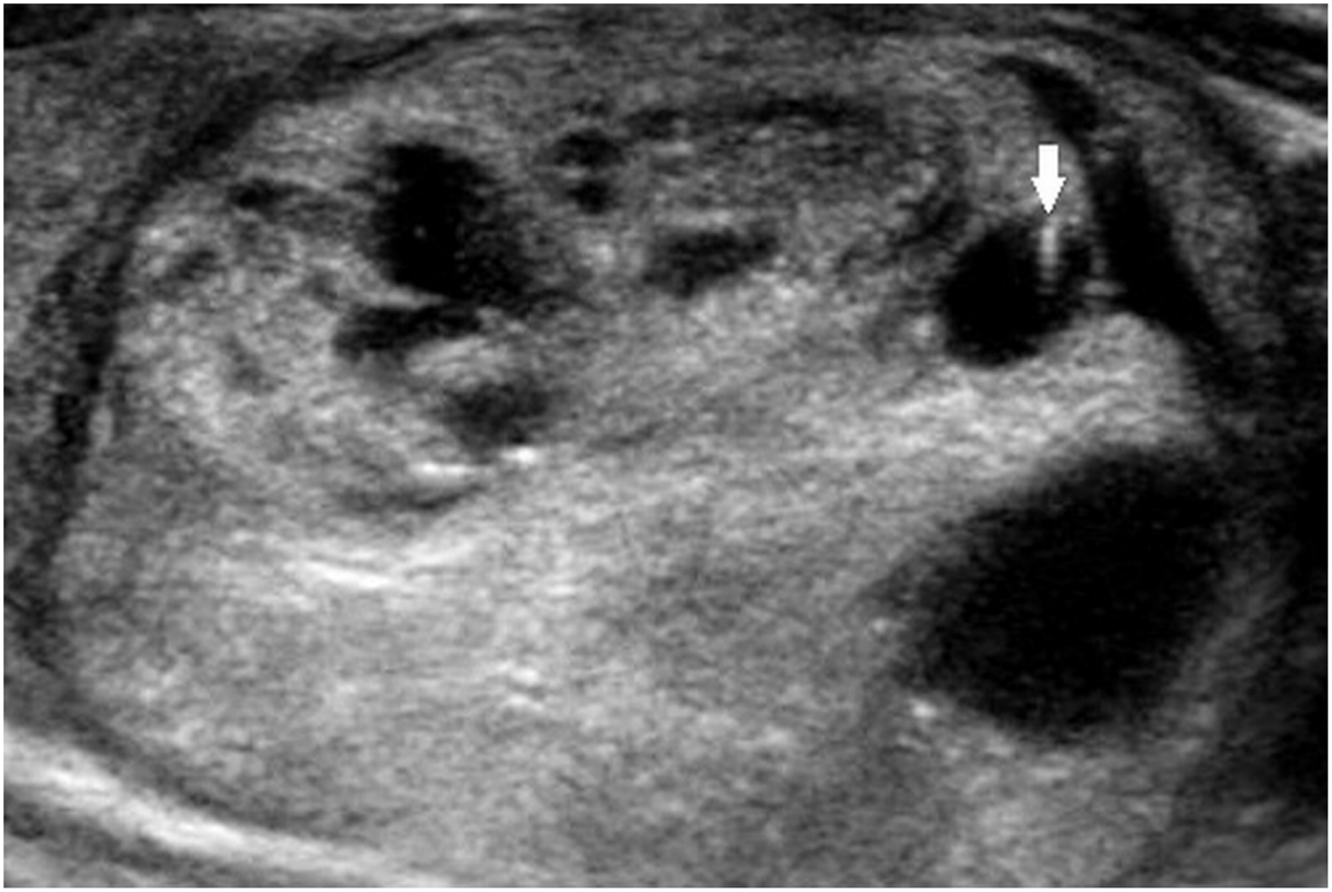
A 41-year-old man with multinodular goiter. Longitudinal sonogram shows an 36-mm, smooth, mixed isoechoic nodule in the right thyroid lobe. Echogenic focus is located at the margin of solid component (solid arrow); comet-tail artifact is located within the cystic component (Type 2).

**Fig 3:**
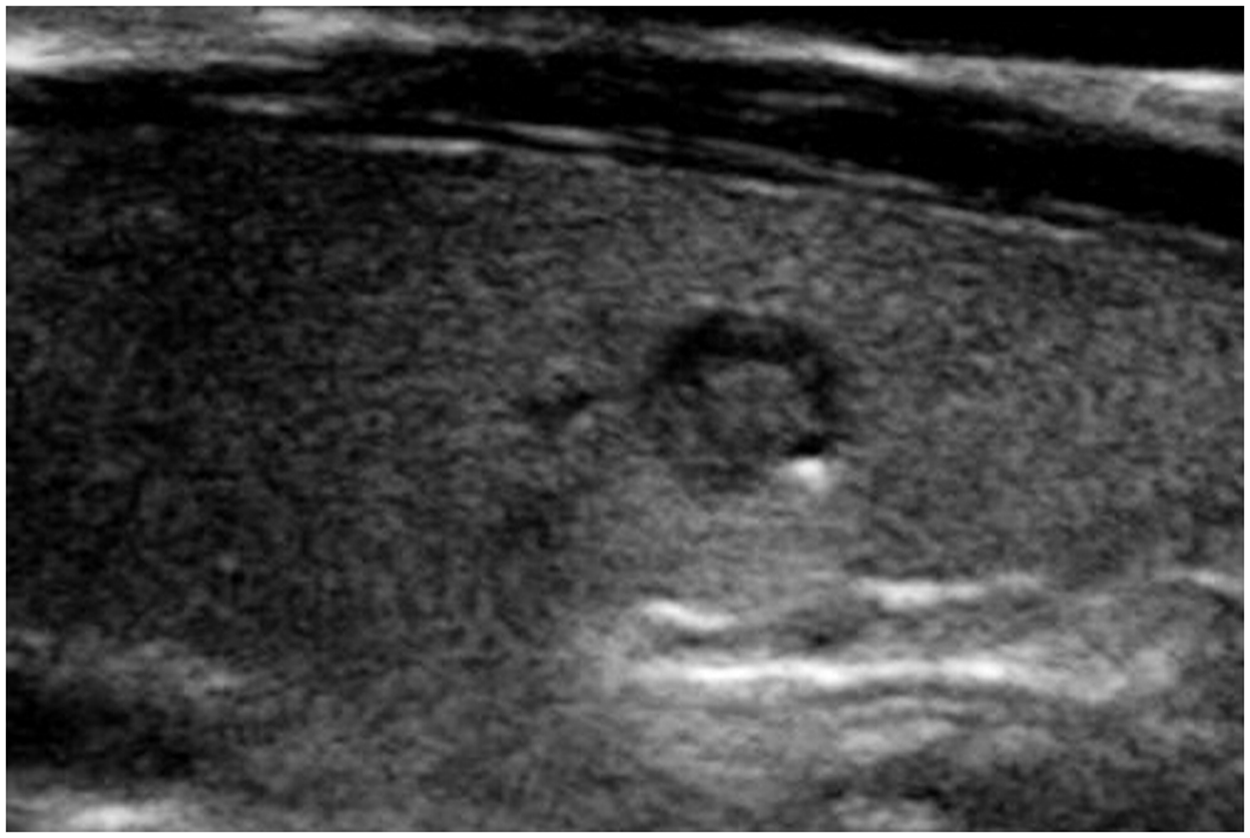
A 31-year-old woman with papillary thyroid carcinoma. Longitudinal sonogram shows a 5-mm, smooth, solid hypoechoic nodule in the right thyroid lobe. The echogenic focus (solid arrow) is located at the margin of intra-nodular micro-cyst. Comet-tail artifact was detected in the solid component (Type 2).

**Fig 4:**
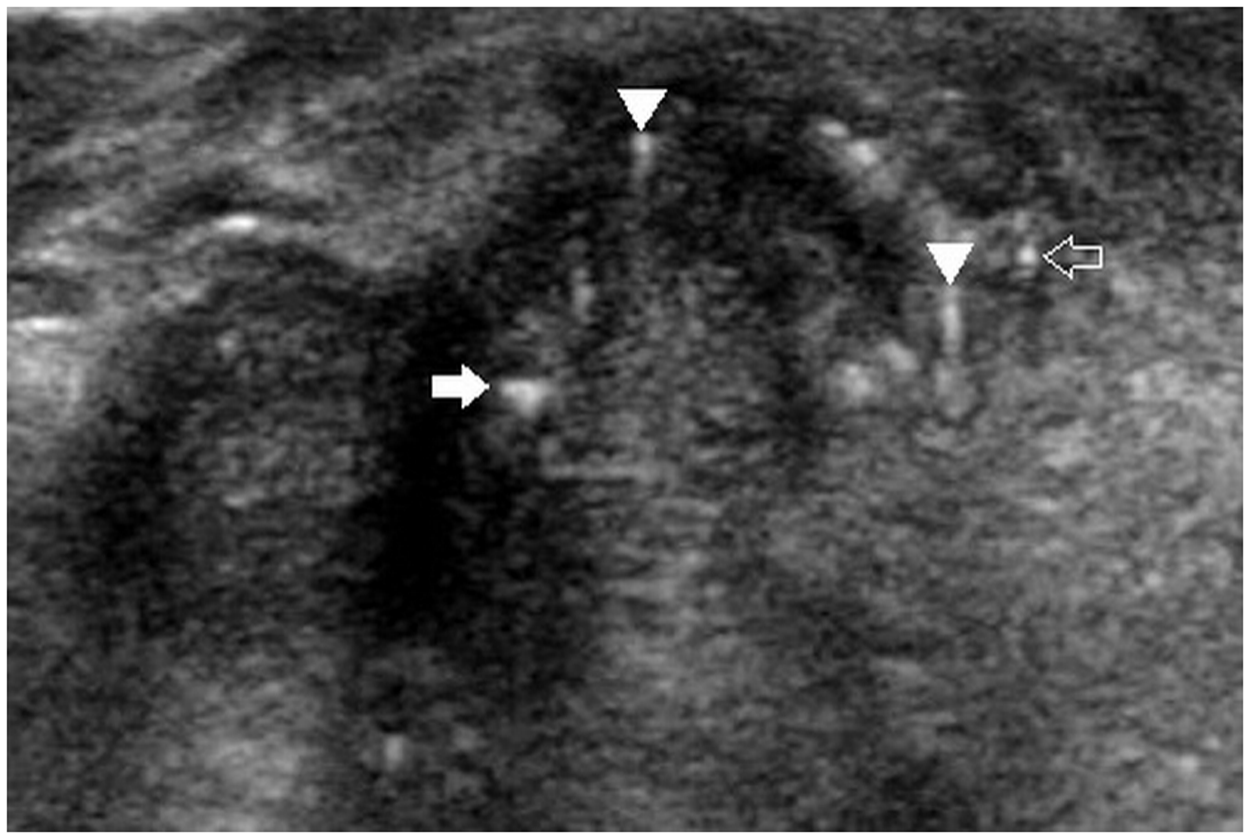
A 52-year-old woman with papillary thyroid carcinoma. Longitudinal sonogram showing a 45-mm, ill-defined, hypoechoic solid nodule in the right thyroid lobe. A linear echogenic focus with reverse-triangle artifact (solid arrow), and a round echogenic focus with fine artifact (arrowhead), and a punctate echogenic foci (faint arrow) are seen (Type 3).

Two radiologists with 19 and 8 years of experience in thyroid imaging, who was blinded to the final pathological diagnosis, retrospectively reviewed sonographic data. Final decisions were reached by consensus. First, the nodules that contained echogenic foci with comet-tail artifact were screened out on a review of ultrasound reports. Corresponding sonographic images were reviewed to assess the sub-type of echogenic foci. With regard to background, echogenic foci with comet-tail artifact were categorized into 3 subtypes: type 1, echogenic foci accompanied by comet-tail artifact were freely distributed in the cystic component (Fig 1); type 2, intermediate type, included: echogenic foci were located at the margin of solid components while comet-tail artifact was located in the cystic component (Fig 2), or echogenic foci were located at the margin of cystic components, while the comet-tail artifact located in the solid component (Fig 3). In brief, echogenic foci in type 2 are associated with solid component as well as cystic component; and type 3, both echogenic foci and comet-tail artifact located in the solid component (Fig 4). If a nodule presented several types of echogenic foci, each echogenic focus was documented separately. The appearance of echogenic foci was categorized as round or linear. For each individual nodule, if echogenic focus within the same background showed different appearances, the predominant appearance was recorded. The shape of comet-tail artifact was classified into two types: a typical appearance of reverse triangle and fine (linear, not reverse triangle) reverberation.

### Statistical analysis

Statistical analyses were performed with SAS version 9.1 software for Windows (SAS Institute Inc, Cary, NC). The χ^2^ (Chi squared) test or Fisher’s exact test was used. A *P* value of less than 0.05 was considered as statistically significant.

## Results

The baseline characteristics of the study participants are showed in Table 1. A total of 71 (71/962, 7.4%) nodules in 556 patients were noted to have echogenic foci with comet-tail artifact; 46 nodules were found to be malignant and 25 as benign by pathology. Malignant nodules included 43 conventional papillary thyroid carcinomas (3 nodules with sporadic cystic portion), 1 cystic papillary thyroid carcinoma (cystic portion > 50%), one follicular thyroid carcinoma and one medullary thyroid carcinoma. Benign thyroid nodules included 22 nodules with multinodular goiter and 3 nodules with adenomatous hyperplasia. Thirty-three malignant nodules and all benign nodules showed colloid at histopathological examination.

**Table 1:** Baseline characteristics of study participants (n = 63).

Variables	No. or median value (range)
Age (years)	44 (20–70)
Gender, male/female	16/47
Nodule number, solitary/multiple	19/44
Main (or maximum) nodule size (mm)	18 (6–47)
Laboratory tests	
Thyroid function, normal/abnormal	55/8
Thyroglobulin antibody, negative/positive	50/13
Thyroid peroxidase antibody, negative/positive	47/16
Family history	
Benign	14
Malignant	3
None	26
Unknown	20

In the background assessment, 66 nodules had one unique appearance. Four nodules had two individual appearances, including 2 benign nodules (type 1 & 2; type 1 & 3, respectively) and 2 malignant nodules (both have type 2 & 3). One benign nodule had three individual appearances. With regard to background, none of the malignant nodule had type 1, echogenic foci with comet-tail artifact of type 2 or type 3 were present in 48 (100%) malignant nodules and 19 (63%) benign nodules (*P* <0.001).

Although benign nodules presented a linear appearance more frequently than that of malignant nodules (15.4% *vs*. 4.3%), there was no significant difference between the benign and malignant nodules with regard to the shape of the echogenic foci (*P* = 0.139). The presence of reverse-triangle was less frequent than that of fine reverberation in both the groups, and there was no statistical difference in terms of the shape of comet-tail between benign and malignant nodules (*P* = 0.626). Table 2 shows the number of sonographic findings of echogenic foci with comet-tail artifact and the pathologic results.

**Table 2:** Distribution of sonographic findings of 78 echogenic foci with comet-tail artifact in 71 nodules compared with pathological results.

Findings		Benign	Malignant	
		n = 30	n = 48	
Background				
	Type 1	11	0	*P* <0.001
	Type 2	8	3	
	Type 3	11	45	
Shape	Round	26	46	*P* = 0.139
	Linear	4	2	
Comet-tail	Reverse-triangle	2	2	*P* = 0.626
	Fine	28	46	

## Discussion

The significance of echogenic foci in thyroid imaging has long been a subject of research, the understanding of which continues to evolve. At the early stage, a common pitfall in sonographic interpretation is to mistake punctate echogenic foci for microcalcification, a type of echogenic foci without posterior acoustic shadowing. The latter is reported to probably be a sonographic representation of psammoma bodies at histological examination, and a specific finding associated with papillary thyroid carcinoma [6, 12]. However, comet-tail artifact with a characteristic reverse triangular shape, an artifact caused by the principle of reverberation, was found associated with a number of echogenic foci at thyroid imaging [13]. Based on a review of 100 thyroid nodules, which varied from purely cystic nodules to complex cystic nodules, Ahuja *et al*. reported that the presence of comet-tail artifact in the cystic component is a useful indicator of benignity with a sensitivity and specificity of 100% [7]. Reading *et al*. also had identified this pattern as a classic appearance indicative of benign thyroid nodular hyperplasia [8]. Subsequently, Hoang *et al*. defined this specific appearance as inspissated colloid calcification, which is distinguishable from malignant calcification by the presence of ring-down or reverberation artifact [6]. Gradually, medical professionals have learnt to avoid mistaking microcalcification for inspissated colloid calcification, by recognition of the comet-tail artifact.

But recent reports suggest that comet-tail artifact could be associated with thyroid malignancy as well. Malhi *et al*. reported that irrespective of its length, the presence of comet-tail was associated with malignancy (range, 3.9–15.4%) [10]. Although punctate echogenic foci with comet-tail artifact were associated with other morphologic types, Tahvildari *et al*. found that 5 of 29 papillary thyroid carcinomas (17%) showed at least 1 site of comet-tail artifact [14]. Similar cases have been reported elsewhere [15–16]. None of these studies addressed the risk for malignancy when echogenic foci with comet-tail artifact associated with solid components in resected thyroid nodules with echogenic foci. In our study, 61.5% (48/78) of echogenic foci were associated with malignancy. We classified echogenic foci with comet-tail artifact into 3 subtypes and found that any type of comet-tail artifact associated with solid component represents a risk of malignancy in 27.3% (3/11, type 2) to 80.4% (45/56, type 3) of cases. When associated with cystic components, the artifact is almost always benign. The main reason of discrepancy compared with previous studies was probably due to the inclusion population. It also reminded us to reexamine the significance of echogenic foci with comet-tail artifacts in specific thyroid nodules.

Klang *et al*. had suggested that echogenic foci with a comet-tail artifact associated with the solid component are more indicative of microcalcifications [16]. In this study, we found a papillary thyroid carcinoma with a comet-tail artifact located in the solid component, which probably caused by posterior reverberation of micro-cystic change, or echogenic foci at the margin of the cystic component. As shown by Tahvilari *et al*., colloid could be detected in all malignant nodules at histological examination [14]. Ginat *et al*. reported colloid was identified on both ultrasound and cytology in 25% of malignant nodules [17]. Hence we can see that colloid is not just limited to benign lesions, but may also exist in malignant lesions. We believe that colloid calcification is liable to be misinterpreted as microcalcification in the absence of comet-tail artifact. Predictably, finer micro-cystic changes related to colloid, regardless of comet-tail artifact, are undetectable using ultrasound at the currently used resolution. Based on the corresponding inference, posterior wall enhancement manifest as echogenic foci mimicked microcalcification of papillary carcinoma. Therefore, we believe that even in the absence of micro-cystic changes, echogenic foci with comet-tail artifact are not the same as microcalcification. Similarly, for those within the solid component, not all echogenic foci without comet-tail artifact are microcalcification. This needs to be confirmed in prospective studies. Moreover, the recognition of artifact greatly depends on technical factors, for instance, adjusting the focal zone could enhance or weaken the artifact [13].

Punctate echogenic foci may correlate with any calcified deposition on histological examination, including collagenous structures, foreign bodies, amyloid, inspissated colloid calcification, dystrophic calcification and psammoma bodies [18]. Psammoma bodies are identified in up to 50% of papillary thyroid carcinomas and have been reported in some benign non-neoplastic conditions, for instance, in Hashimoto’s thyroiditis and nodular goiter with papillary hyperplasia [19–20]. Spherical calcified focus showing concentric laminations and located within stromal stalks of tumor papillae, but not within the lumen of a follicle can just define as a psammoma body [21]. The specific microscopic representation is related to the origin of psammoma bodies, which are formed by focal areas of infarction of the tips of papillae, presumably due to thrombosis of the delicate vessels or damage from minor trauma [22]. Analysis of the available literature shows that, with regard to location and association, psammoma bodies can be distinguishable from inspissated colloid calcification, as the latter is found within the follicle lumen, lacks concentric laminations. Of the seventy-two echogenic foci with round shape in this study, 26 (36.1%) were present in benign nodules. In contrast, echogenic foci with linear shape (4/6, 66.7%) were found much more frequently in benign than malignant nodules. We hypothesize that the difference in sonographic imaging is partly associated with the morphological diversity at histopathologic examination. It is possible for psammoma bodies to fuse together, making larger concretions, and thereby lose their spherical form. The echogenic foci in linear shape at sonographic imaging may represent aggregation of multiple calcified deposits on histopathological examination. Future study on precise radiologic-cytologic correlation is needed to validate the importance of shape in the differential diagnosis not only of echogenic foci with comet-tail artifact, but also punctate echogenic foci.

This study has some limitations. First, it is a relatively small retrospective case series; only the nodules with histopathological results were included in the analysis, which may not be representative of the epidemic of thyroid nodules and likely introduced an element of selection bias which may have potentially inflated the malignant rate. In order to rule out confounding factors, patients with clinical or ultrasound follow-up results, i.e. more benign nodules, were excluded. Selection bias also should respond to the discordance that echogenic foci with comet-tail artifact were seen in only 7.4% of nodules in this study, it is much less than that of Malhi et al. (up to 29% for small comet-tail). Second, we did not review the real-time imaging but rather static imaging. Although parts of information were recorded in the dynamic study, the interobserver variability with respect to interpretation of each individual radiologist could not be assessed because of the retrospective nature of the analysis and methological limitations of the study. Third, the interobserver variability in the interpretation of the US findings based on two radiologists wasn’t available.

## Conclusion

We examined the echogenic foci in 71 resected thyroid nodules and investigated its clinical significance. There was a significant association between background of echogenic foci and histopathological results in resected thyroid nodules. Echogenic foci with comet-tail artifact freely distributed in cystic component may predict a benign nodule; those associated with solid components cannot be considered a benign finding. We propose not to interpret the presence of echogenic foci with comet-tail artifact as synonymous with benignity in generalities.
